# KNEE OA AS A SYSTEMIC MODEL OF AGING

**DOI:** 10.1093/geroni/igac059.2447

**Published:** 2022-12-20

**Authors:** Dennis Lox

**Affiliations:** Clearwater Fl, Clearwater, Florida, United States

## Abstract

Knee osteoarthritis (OA) and aging, may be viewed differently by various patients and health care providers. Aging is now considered as a systemic interplay of molecular, cellular, tissue, hormonal, and body dysfunctions. Important drivers of these factors lead to osteoarthritis, coronary artery disease, cancer and dementia. An understanding of each as its progresses can alter morbidity. The proposed theories of aging hold true for knee OA. A simple joint compartment model, such as the knee, may help this understanding. A degenerative knee OA joint is a progressive disorder, just like aging. Knee OA may be accelerated by trauma, aging, decreased autophagy, cellular changes, cytokine production, disruption of the matrix, and cellular senescence. The associated SASP leads to the progressive cascade of degenerative changes. Understanding how to mitigate these affects provides a framework for a longer healthspan. Obesity, and it’s adipokines exacerbate the inflammation, and compound weigh bearing stress. Promotion of longevity pathways, stimulate repair via Sirt, AMPK, FOXO and decreased stimulation of MTor, and FOXO4- P53 coupling. The resulting changes can altering the nature of the accelerated nature of progressive knee OA. Joint conservation, unregulated IL-4, IL-10, IRAP, and down regulation of NF-kB, and the ensuing cascade of HMBG1, and DAMP. Hippocrates wrote walking is man’s best medicine, however, with knee OA this may need amendment, to encourage proper medical guidance for appropriate exercise and diet. It is time to merge knee OA and aging models to work in harmony for prolonged health span.

